# A four-gene signature predicts survival in clear-cell renal-cell carcinoma

**DOI:** 10.18632/oncotarget.12631

**Published:** 2016-10-13

**Authors:** Jun Dai, Yuchao Lu, Jinyu Wang, Lili Yang, Yingyan Han, Ying Wang, Dan Yan, Qiurong Ruan, Shaogang Wang

**Affiliations:** ^1^ Cancer Biology Research Center, Tongji Hospital, Tongji Medical College, Huazhong University of Science and Technology, Wuhan, China; ^2^ Department and Institute of Urology, Tongji Hospital, Tongji Medical College, Huazhong University of Science and Technology, Wuhan, China; ^3^ Department of Orthopaedics and Traumatology, Prince of Wales Hospital, The Chinese University of Hong Kong SAR, Hong Kong, China; ^4^ Institute of Pathology, Tongji Hospital, Tongji Medical College, Huazhong University of Science and Technology, Wuhan, China; ^5^ Department of Pathology, Medical College, Wuhan University of Science and Technology, Wuhan, China

**Keywords:** ccRCC, prognosis, gene signature, tissue microarray, bioinformatics

## Abstract

Clear-cell renal-cell carcinoma (ccRCC) is the most common pathological subtype of renal cell carcinoma (RCC), accounting for about 80% of RCC. In order to find potential prognostic biomarkers in ccRCC, we presented a four-gene signature to evaluate the prognosis of ccRCC. SurvExpress and immunohistochemical (IHC) staining of tissue microarrays were used to analyze the association between the four genes and the prognosis of ccRCC. Data from TCGA dataset revealed a prognostic prompt function of the four genes (PTEN, PIK3C2A, ITPA and BCL3). Further discovery suggested that the four-gene signature predicted survival better than any of the four genes alone. Moreover, IHC staining demonstrated a consistent result with TCGA, indicating that the signature was an independent prognostic factor of survival in ccRCC. Univariate and multivariate Cox proportional hazard regression analysis were conducted to verify the association of clinicopathological variables and the four genes’ expression levels with survival. The results further testified that the risk (four-gene signature) was an independent prognostic factors of both Overall Survival (OS) and Disease-free Survival (DFS) (*P*<0.05). In conclusion, the four-gene signature was correlated with the survival of ccRCC, and therefore, may help to provide significant clinical implications for predicting the prognosis of patients.

## BACKGROUND

Renal cell carcinoma (RCCs) is derived from the renal tubule epithelium, and is one of the most common malignant tumors in the urinary system. Approximately 90% of renal tumors that are diagnosed in adults are RCCs, and the most common pathological subtype is ccRCC, which accounts for about 80% of RCCs [1, 2]. In recent years, the incidence and mortality of renal cell carcinoma have been increasing worldwide [3]. Currently, less than 6% to 10% of the patients appear in the clinical practice with the typical triad (i.e., hematuria, back pain, and abdominal mass) [4]. Moreover, tumor invasion and metastasis is the leading cause of death in RCC patients, and nearly 25% of patients have metastasis when they come to treatment [5]. The most common site of metastasis is the lung, followed by the liver, bone, and brain, as well as the contralateral kidney.

Although great progress has been made in molecular biology research on the pathogenesis of RCCs, nephrectomy remains to be the main therapy. Researchers have found that only early stage ccRCC (T1-2) can be treated with surgery and may have a good long-term prognosis [6]. For metastatic and late-stage ccRCC (T3-4), the curative effect of chemoradiotherapy and surgery are poor [7, 8]. Problematically, molecular-targeted therapies, such as axitinib, sorafenib and temsirolimus, have an efficiency of only 10% to 40%[9]. Similarly, immunotherapy also has a low efficacy. Regardless of the therapeutic strategy, the long-term outcome is poor for most ccRCC patients. Therefore, investigation on the molecular mechanism of ccRCC is necessary to better understand the behavior of the disease, predict the prognosis, inform rational treatment programs, and provide novel therapeutic targets.

Many genes have been reported to be involved in the tumorigenesis and progression of the tumor, and have been found to be correlated with patient prognosis and survival. For example, phosphatase and tensin homologue deletion on chromosome 10 (PTEN) is one of the most frequently mutated human tumor suppressor genes [10]. PTEN is located on human chromosome 10q23.3, and encodes a protein containing 403 amino acids. It functions as a dual protein and lipid phosphatase and has been reported to inhibit cell growth and survival, suggesting a critical tumor suppressor effect [10, 11]. In recent years, many studies have shown that PTEN often has an abnormal frequency of deletions, genetic mutations or methylation in a variety of cancers, such as prostate cancer and renal cell carcinoma [11–14]. In addition, PTEN has been found to be closely related to the tumor metastasis and invasion. Loss of PTEN can also result in abnormal activation of the Phosphatidyl Inositol 3-kinase/Protein Kinase B (PI3K/Akt) pathway, which regulates proliferation, apoptosis, survival, translation, differentiation and cellular metabolism [15].

The PI3K/Akt pathway can also become activated by the upregulation of kinases in the pathway, such as phosphatidylinositol-4-phosphate 3-kinase catalytic subunit type 2 alpha (PIK3C2A), which belongs to the class II PI3Ks and plays an essential role in angiogenesis [16, 17]. In fact, upregulation of PIK3C2A has been reported in several cancers [18], such as breast cancer, cervical cancer, lung cancer, stomach cancer, colon cancer, liver cancer and oral squamous cell carcinoma [19].

Inosine triphosphate pyrophosphohydrolase (ITPA) is an enzyme that is involved in the 6-Mercaptopurine metabolic pathway and is responsible for converting inosine triphosphate (ITP) back to inosine monophosphate (IMP), thereby preventing the accumulation of the toxic metabolite ITP. In recent years, ITPA has been reported to be one of the five mixed-lineage leukemia associated genes and its upregulation may lead to amplification of the Mixed Lineage Leukemia (MLL) gene region of 11q23 [20]. ITPA expression is also associated with event-free survival and relapse rates in children with acute lymphoblastic leukemia that are undergoing maintenance therapy [21]. Further evidences show that the absence of functional ITPA activity can result in elevated mutagenesis and accumulation of non-canonical nucleotides, which may cause DNA damage and cancer, indicating a significant role of ITPA in preventing base analog-induced apoptosis, DNA damage and mutagenesis in human cells [22]. Conversely, overexpression of ITPA has been reported in various cancer cell lines, such as colon, lung, liver, pancreatic, and brain [23]. In addition, the expression level of ITPA is higher in stage III melanoma patients with poor prognosis, compared with patients having a good prognosis [24].

B-cell lymphoma 3 (BCL3) is a proto-oncogene that belongs to the Iκ-B family. It has been pointed out to be upregulated in hematological malignancies, as well as in a wide range of solid tumors [25, 26], including breast cancer, ovarian cancer, colorectal cancer and non-small-cell lung cancer, and it is also associated with the survival and relapse frequency [27–29]. Furthermore, BCL3 has been reported to exhibit anti-apoptotic effect in cancer [30, 31], which plays a proto-oncogene role.

However, no study had been reported to clarify the relationship between the four genes (PTEN, PIK3C2A, IPTA and BCL3) together and diseases. Of course, some reports had revealed links between paired comparisons of the four genes. For example, PI3K/PTEN expression was frequently deregulated in many malignancies contributing to the upregulation of PI3K/Akt/mTOR pathway. The activation of PI3K/PTEN/Akt/mTOR pathway was implicated in both the pathogenesis of malignancies and the resistance to anticancer therapies [32]. BCL-3 was reported to increase in human colorectal cancers and can promote cell survival under tumor microenvironment. It may protect colorectal adenoma/carcinoma cells from apoptosis though activation of AKT pathway, which was mediated by PI3K/mTOR pathways [33].

However, analyses on the association between the four genes’ expression and survival in ccRCC patients remain limited. In this study, *SurvExpress* analysis, tissue microarrays and IHC techniques were used to detect the expression of PTEN, PIK3C2A, ITPA and BCL3 in ccRCC, and to explore their relationship with survival. Our findings may provide valid indicators for clarifying the pathogenic mechanism of ccRCC and predicting the prognosis.

## RESULTS

### Survival analysis with *SurvExpress*

**Figure 1:**
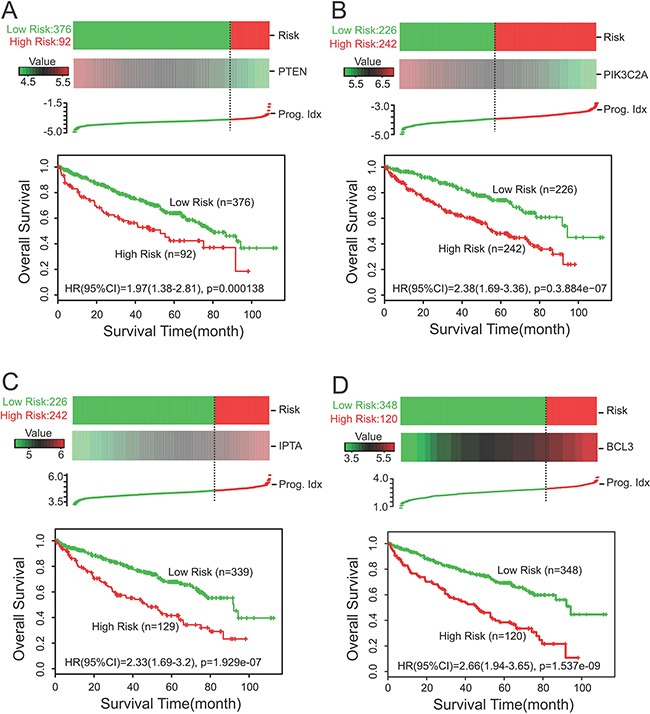
Survival analysis with SurvExpress (*n*=468) **A, B.** Low expression of PTEN and PIK3C2A were correlated with high risk, poor prognosis and shorter OS time. **C, D.** High expression of ITPA and BCL3 indicated high risk, poor prognosis and shorter OS time. Kaplan-Meier survival curves were also constructed to reveal the relationship between predicted risk of ccRCC patients and the OS time. The results showed that patients with high risk had a significantly shorter OS time than those with low risk (A-D). Green and red lines indicated low- and high-risk groups, respectively. *P* <0.05 was considered to be statistically significant. Cens: Censored; Event: Death; Prog. Idx.: Prognosis Index.

We analyzed PTEN, PIK3C2A, ITPA and BCL3 expression in TCGA dataset (KIRC) from *SurvExpress*. The patients from TCGA (*n* = 468) were classified into predicted low and high risk groups according to the Prognostic Index (PI). The results demonstrated that low expression of PTEN and PIK3C2A were significantly correlated with high risk, poor prognosis and shorter OS time (Figure 1A, 1B), while high expression of ITPA and BCL3 indicated high risk, poor prognosis and shorter OS time (Figure 1C, 1D). Moreover, survival differences between predicted low and high risk groups were evaluated with Kaplan-Meier survival curves. Our results showed that patients with high risk had a significantly shorter OS time than those with low risk (Figure 1). Green and red lines indicated low- and high-risk groups, respectively. *P* <0.05 was considered to be statistically significant

### Expression of PTEN, PIK3C2A, ITPA and BCL3 in ccRCC and their relationship with survival

**Figure 2:**
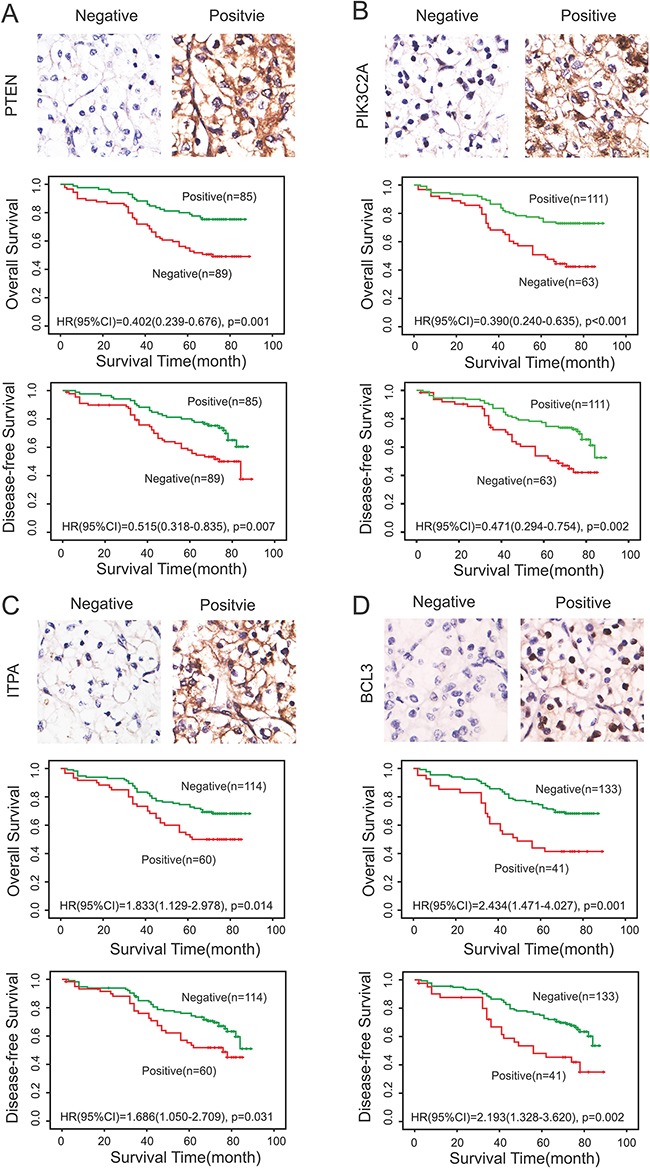
Expression of PTEN, PIK3C2A, ITPA and BCL3 in ccRCC and their relationship with OS and DFS IHC analysis on tissue microarrays and Kaplan-Meier survival curves were constructed to verify the relationship between PTEN **A.**, PIK3C2A **B.**, ITPA **C.** and BCL3 **D.** expression with regard to OS and DFS. (A, B) Negative expression of PTEN and PIK3C2A were correlated with shorter OS and DFS time and worse prognosis. (C, D) Positive expression of ITPA and BCL3 were correlated with shorter OS and DFS time and worse prognosis. Green and blue lines indicated positive and negative expression groups, respectively. *P* <0.05 was considered to be statistically significant.

To verify the relation between PTEN, PIK3C2A, ITPA and BCL3 expression with regard to survival and risk, we first performed IHC analysis on tissue microarrays. The expression levels of the four proteins were all divided into two groups (negative and positive expression groups) based on the staining score. A total score of 0–4 points was defined as negative expression, whereas 5–6 points were considered as positive expression. Our study showed that the positive expression rate of PTEN, PIK3C2A, ITPA and BCL3 in 174 cases of ccRCC were 48.9% (Figure 2A), 63.8% (Figure 2B), 34.5% (Figure 2C) and 23.6% (Figure 2D), respectively. Furthermore, Kaplan-Meier survival curves were constructed to analyze the relationship between PTEN, PIK3C2A, ITPA and BCL3 expression and OS as well as DFS. A consistent result with that from the TCGA dataset was shown. Our results demonstrated that negative expression of PTEN and PIK3C2A were correlated with shorter OS and DFS time and worse prognosis (Figure 2A, 2B), while positive expression of ITPA and BCL3 were correlated with shorter OS and DFS time and worse prognosis (Figure 2C, 2D).

### The four-gene signature predicted survival in ccRCC

**Figure 3:**
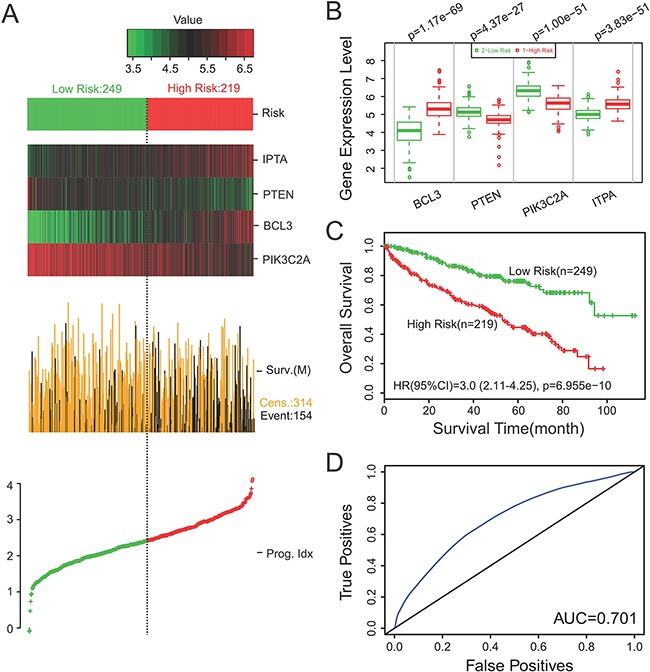
The four-gene signature predicted survival better than the individual genes alone in ccRCC **A.** *SurvExpress* was used to analyze the association of the four-gene signature with the predicted risk, survival time and prognosis. **B.** The gene expression level of PTEN, PIK3C2A, ITPA and BCL3 were detected in high risk and low risk group. **C.** Kaplan-Meier survival curves showed that patients with predicted high risk (*n* = 219) had significantly shorter OS time than those with low risk (*n* = 249) (*P* <0.05). **D.** ROC analysis was performed to compare the sensitivity and specificity of the survival prediction between our models. *P* <0.05 was considered to be statistically significant. Cens: Censored; Event: Death; Prog. Idx.: Prognosis Index; Sur.(M): Survival status (Month).

Using this four-gene signature, we analyzed its ability to predict survival using TCGA with *SurvExpress*. In our four-gene signature, the PI of the 468 patients was from -0.1001 to 4.1239, with the optimal cut-off value of 2.43. PI that less than 2.43 was divided into low risk group (n = 249), while PI that higher than 2.43 was High Risk group (n = 219). The analysis demonstrated that high risk was correlated with low expression of PTEN and PIK3C2A, high expression of ITPA and BCL3, shorter survival time and worse prognosis, while low risk was correlated with high expression of PTEN and PIK3C2A, low expression of ITPA and BCL3, longed survival time and better prognosis (Figure 3A). Moreover, we detected the gene expression level of PTEN, PIK3C2A, ITPA and BCL3 in high risk and low risk group. Our results displayed that the gene expression of PTEN and PIK3C2A were lower in high risk group than that in low risk group, while the gene expression of ITPA and BCL3 were higher in high risk group than that in low risk group, and all had significant difference in the four-gene signature (*P* = 4.37e-27, *P* = 1.00e-51, *P* = 3.83e-51 and *P* = 1.17e-69, respectively) (Figure 3B). Moreover, Kaplan-Meier survival curves showed that patients with predicted high risk (*n* = 219) had significantly shorter OS time than those with low risk (*n* = 249) (*P* <0.05) (Figure 3C). To estimate the accuracy of the four-gene signature on predicting survival, we performed receiver operating characteristic (ROC) analysis to compare the sensitivity and specificity of the survival prediction between our models. TCGA dataset revealed that the area under receiver operating characteristic (AUC) curve of the four-gene signature was 0.701 (time = 60 months) (*P*<0.05) (Figure 3D).

**Figure 4:**
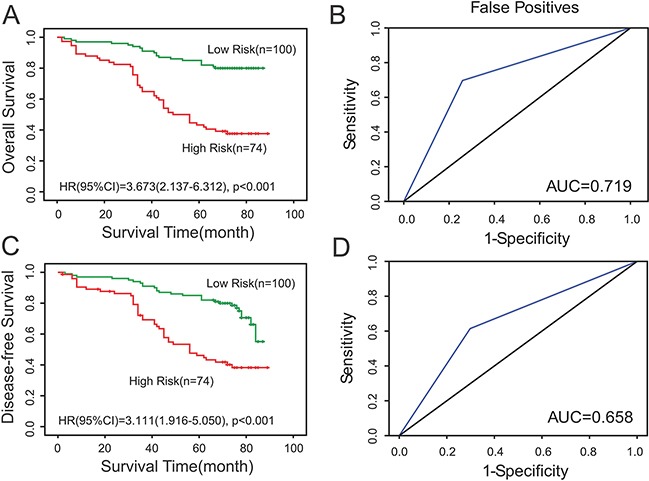
Prediction of the prognosis of ccRCC patients by the four-gene signature on tissue microarrays **A, C.** Kaplan-Meier survival curves indicated shorter DFS and overall OS time for the high-risk group (*n* =74) than in the low-risk group (*n* =100) (*P* <0.05). **B, D.** ROC analysis was performed to compare the sensitivity and specificity of the models. *P* <0.05 was considered to be statistically significant.

In addition, the 174 patients of ccRCC were also divided into two groups (low risk group and high risk group) based on IHC of the four proteins. Patients that coincided with at least three of PTEN (+), PIK3C2A (+), ITPA (-) and BCL3 (-) were characterized as low risk group, while the remaining was considered as high risk group. Consistent with the above results, Kaplan-Meier survival curves also indicated shorter OS and DFS time for the high-risk group (*n* =74) than in the low-risk group (*n* =100) (*P* <0.05) (Figure 4A, 4C). ROC analysis was also performed to compare the sensitivity and specificity of the models. Our data showed an AUC of 0.719 in OS model and 0.658 in DFS model (*P*<0.05) (Figure 4B, 4D). The specificity and sensitivity of the four-gene signature were 0.697 and 0.741 respectively in Overall survival analysis, while they were 0.614 and 0.702 respectively in Disease-free survival analysis.

### Correlation between clinicopathological features and the expression of PTEN, PIK3C2A, ITPA and BCL3 in ccRCC

Table 1 lists the relationship between clinicopathological features and the expression of PTEN, PIK3C2A, ITPA and BCL3 as well as Risk in the 174 cases of ccRCC patients. Our study revealed that the expression of BCL3 (*P* = 0.028) and Risk (four-gene signature) of the patients (*P* = 0.040) were significantly correlated with the clinical grade of the tumors. Moreover, the classification of Risk based on the four-gene signature (*P* = 0.006) was significantly related with the size of the tumors. Furthermore, our data suggested that the expression of BCL3 and ITPA may be associated with PTEN expression (*P* = 0.012, *P* = 0.002, respectively).

**Table 1:** Correlation between clinicopathological features and the four proteins expression in ccRCC (*n* =174)

	PTEN		*P*	BCL3		*P*	ITPA		*P*	PIK3C2A		*P*	Risk		*P*
	Negative	Positive		Negative	Positive		Negative	Positive		Negative	Positive		Low	High	
Gender															
Male	51	44	0.463	73	22	0.890	62	33	0.938	39	56	0.145	51	44	0.268
Female	38	41		60	19		52	27		24	55		49	30	
Age															
≤55	30	30	0.826	47	13	0.669	43	17	0.216	24	36	0.450	36	24	0.625
>55	59	55		86	28		71	43		39	75		64	50	
Grade															
1,2	66	63	0.995	104	25	0.028*	88	41	0.205	44	85	0.329	80	49	0.040*
3,4	23	22		29	16		26	19		19	26		20	25	
Size															
≤7	65	64	0.734	102	27	0.166	83	46	0.581	19	40	0.754	82	47	0.006*
>7	24	21		31	14		31	14		11	20		18	27	
Stage															
I,II	77	74	0.916	116	35	0.759	98	53	0.661	26	58	0.073	91	60	0.56
III,IV	12	11		17	6		16	7		4	2		9	14	
PTEN															
Negative				61	28	0.012*	68	21	0.002*	31	58	0.699			
Positive				72	13		46	39		31	53				
BCL3															
Negative							88	45	0.746	46	87	0.423			
Positive							26	15		17	24				
ITPA															
Negative										40	74	0.672			
Positive										23	37				

Grade: Furhman Grade; *P* value was obtained from the Chi-square test. *P < 0.05 was considered to be statistically significant.

### Survival prediction effect of Risk at different grade in cancer patients

**Figure 5:**
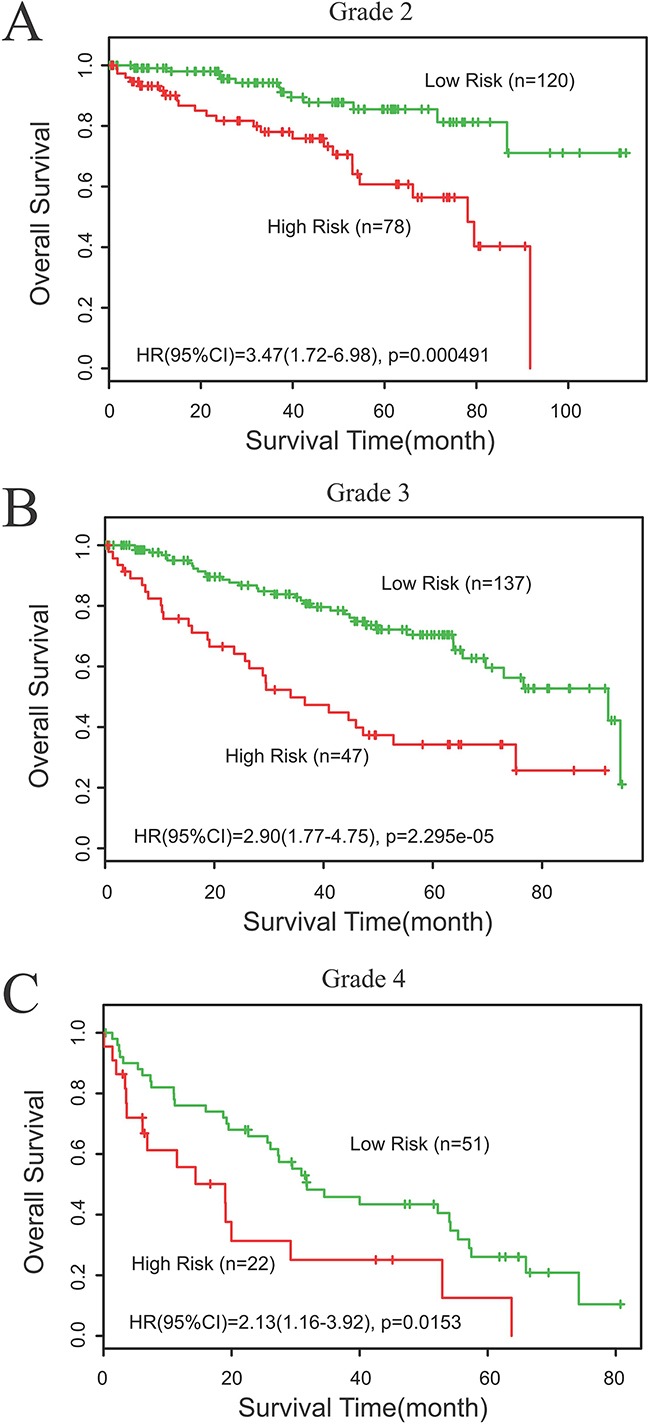
Subgroup analysis of the differentiation grade according to Survexpress **A.** Data from TCGA showed statistical significance in prognosis (OS) of grade 2 ccRCC patients between the low risk and high risk group, p<0.05. **B.** Subgroup analysis according to Survexpress revealed that high risk was related with short OS time in ccRCC patients of grade 3, p<0.05. **C.** Subgroup analysis according to Survexpress revealed that high risk was related with short OS time in ccRCC patients of grade 4, p<0.05.

**Figure 6:**
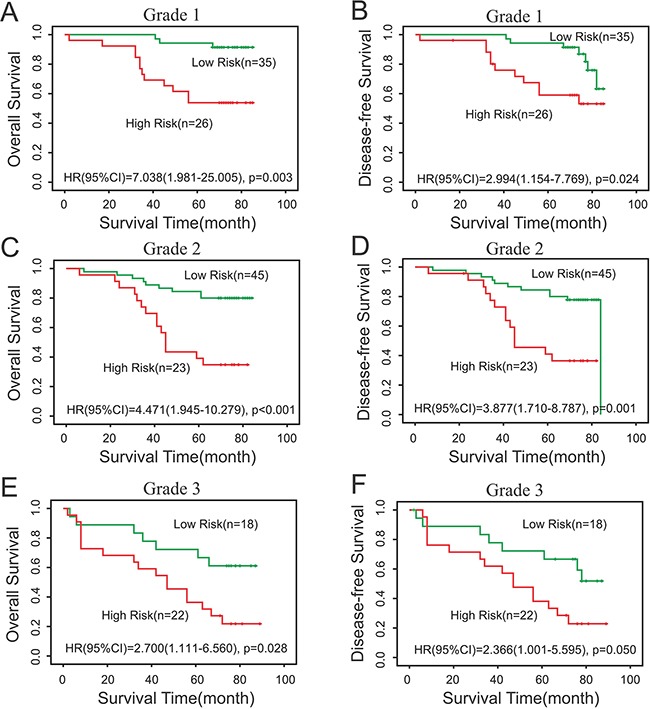
Subgroup analysis of the differentiation grade according to IHC results **A, B.** Subgroup analysis according to IHC results demonstrated that high risk was associated with short OS and DFS time in patients of grade 1, p<0.05. **C, D.** Our survival data revealed statistical significance in prognosis (OS) of grade 2 ccRCC patients between the Risk (low risk and high risk), p<0.05. **E, F.** Our survival data revealed statistical significance in prognosis (OS) of grade 3 ccRCC patients between the Risk (low risk and high risk), p<0.05.

In order to verify whether risk can be used to predict the survival of cancer patients with different grades, we performed subgroup analysis of the differentiation grade according to Survexpress and IHC results. Fuhrman Grade was used for the evaluation of tumor grade. Subgroup analysis according to Survexpress revealed that high risk was all related with short OS time in ccRCC patients of grade 2-4, and showed statistical significance between high risk and low risk group (p<0.05) (Figure 5). Similarly, Subgroup analysis according to IHC results also demonstrated that high risk was associated with short OS and DFS time in patients of grade 1-3, and had statistical significance (p<0.05) (Figure 6).

### Selection of independent prognostic factors for predicting survival in ccRCC

To identify independent factors associated with survival in ccRCC, a univariate Cox proportional hazard regression analysis was conducted to clarify the association of clinicopathological variables and the four genes’ expression levels with OS (Table 2) and DFS (Table 3) in ccRCC. Table 2 revealed that tumor size, grade and clinical stage, the expression levels of the four genes, and risk (four-gene signature) were all correlated with OS. Among the above factors, only PTEN and PIK3C2A were shown to be protective factors in patients, while other indicators that had statistical significance were all risk factors (*P* <0.05). Then, the above-mentioned factors were brought into further multivariate Cox proportional hazard regression analysis. As Risk was evaluated based on the expression level of PTEN, BCL3, ITPA and PIK3C2A, in our opinion, analyzing the Risk and each gene in one multivariate analysis may lead to interference of the results; so two separate multivariate analyses were performed. One multivariate analysis suggested that the expression levels of PTEN, ITPA, PIK3C2A and BCL3 were all independent predictors of OS (*P* <0.05). Another multivariate Cox proportional hazard regression analysis showed that tumor grade and risk were independent prognostic factors (*P* <0.05) (Table 2). Table 3 revealed that tumor size, grade and clinical stage, the expression levels of the four genes, and risk were also correlated with DFS. Similarly, the above-mentioned factors were further brought into two separate multivariate analyses. One multivariate analysis suggested that the expression levels of PTEN, ITPA and PIK3C2A, but not BCL3, were independent predictors of DFS (*P* <0.05). Another multivariate Cox proportional hazard regression analysis showed that only risk (four-gene signature) was an independent prognostic factor (*P* <0.05) (Table 3).

**Table 2:** Univariate and multivariate Cox proportional hazard regression analyses of the association of clinicopathological characteristics and the four genes' expression levels with OS

Characteristics	Univariate		Multivariate		Multivariate	
	HR(95%CI)	*P*	HR(95%CI)	*P*	HR(95%CI)	*P*
Gender(Female vs Male)	1.071(0.660-1.737)	0.781	-	-	-	-
Age (≤55 years vs >55 years)	1.187(0.707-1.993)	0.516	-	-	-	-
Grade (1, 2 vs 3, 4)	2.473(1.513-4.042)	<0.001*	1.625(0.924-2.856)	0.092	1.734(1.006-2.988)	0.047*
Size (≤7cm vs >7cm)	2.321(1.420-3.794)	0.001*	1.695(0.954-3.013)	0.072	1.427(0.815-2.498)	0.213
Stage (I, II vs III, IV)	2.102(1.145-3.858)	0.017*	1.331(0.665-2.663)	0.419	1.127(0.572-2.220)	0.730
PTEN (Negative vs Postive)	0.402(0.239-0.676)	0.001*	0.310(0.177-0.543)	<0.001*	-	-
BCL3 (Negative vs Postive)	2.434(1.471-4.027)	0.001*	1.741(1.024-2.959)	0.040*	-	-
ITPA (Negative vs Postive)	1.833(1.129-2.978)	0.014*	2.553(1.497-4.356)	0.001*	-	-
PIK3C2A (Negative vs Postive)	0.390(0.240-0.635)	<0.001*	0.434(0.264-0.714)	0.001*	-	-
Risk (Low Risk vs High Risk)	4.294(2.534-7.278)	<0.001*	-	-	3.673(2.137-6.312)	<0.001*

Grade: Furhman Grade; HR, hazard ratio; CI, Confidence Interval; *P* Values were obtained from the Chi-square test. * *P* <0.05 was considered to be statistically significant.

**Table 3:** Univariate and multivariate Cox proportional hazard regression analyses of the association of clinicopathological characteristics and the four genes' expression levels with DFS

Characteristics	Univariate		Multivariate		Multivariate	
	HR(95%CI)	*P*	HR(95%CI)	*P*	HR(95%CI)	*P*
Gender(Female vs Male)	0.961(0.600-1.541)	0.870	-	-	-	-
Age (≤55 years vs >55 years)	0.937(0.577-1.522)	0.792	-	-	-	-
Grade (1, 2 vs 3, 4)	2.195(1.354-3.558)	0.001*	1.471(0.844-2.562)	0.173	1.522(0.888-2.609)	0.127
Size (≤7cm vs >7cm)	2.147(1.326-3.477)	0.002*	1.527(0.864-2.697)	0.145	1.353(0.777-2.357)	0.285
Stage (I, II vs III, IV)	2.395(1.367-4.197)	0.002*	1.631(0.847-3.142)	0.143	1.484(0.780-2.825)	0.229
PTEN (Negative vs Postive)	0.515(0.318-0.835)	0.007*	0.420(0.249-0.708)	0.001*	-	-
BCL3 (Negative vs Postive)	2.193(1.328-3.620)	0.002*	1.671(0.984-2.838)	0.057	-	-
ITPA (Negative vs Postive)	1.686(1.050-2.709)	0.031*	2.161(1.295-3.604)	0.003*	-	-
PIK3C2A (Negative vs Postive)	0.471(0.294-0.754)	0.002*	0.512(0.317-0.828)	0.006*	-	-
Risk (Low Risk vs High Risk)	3.111(1.916-5.050)	<0.001*	-	-	2.693(1.637-4.432)	<0.001*

Grade: Furhman Grade; HR, hazard ratio; CI, Confidence Interval; *P* Values were obtained from the Chi-square test. * *P* <0.05 was considered to be statistically significant.

## DISCUSSION

Although great progress has been made in pathogenesis and therapeutic strategy of RCCs, the long-term outcome is poor for most ccRCC patients [34, 35]. Therefore, it is requisite to investigate on the molecular mechanism of ccRCC in order to better understand the disease, predict the prognosis, formulate rational treatment programs, and provide novel therapeutic targets [36]. Wu et al. identified a 4-microRNA (miR-10b, miR-139-5p, miR-130b and miR-199b-5p) signature and it was validated to be associated with ccRCC metastasis and prognosis [37]. Another tumor-specific miRNA signature consisting of 22 miRNAs was also demonstrated as an independent prognostic factor, serving as a novel biomarker for prognostic promopt and treatment outcome prediction in ccRCC [38]. Moreover, Wang et al. revealed that combined chemokine (C-Cmotif) ligand 2 (CCL2) and its receptor CCR2 expression may exert its role as an independent prognostic factor for non-metastatic ccRCC patients after surgical treatment [39].

In the present study, we have identified a four-gene signature (PTEN, ITPA, PIK3C2A and BCL3) that was able to predict ccRCC prognosis for the first time. Each of the four genes we identified had been previously reported to be associated with other types of cancer, as well as survival. However, little was known on the expression and function of these four genes in ccRCC. Moreover, as the efficacy of a single index was limited, multi-biomarker-based model may provide more powerful effect for the prognosis prediction of patients.

In our study, we first analyzed the association of PTEN, PIK3C2A, ITPA, and BCL3 expression with the prognosis of ccRCC patients in TCGA dataset (KIRC) from *SurvExpress*. The data demonstrated that low expression of PTEN and PIK3C2A were significantly correlated with high risk, poor prognosis and a shorter OS time, while high expression of ITPA and BCL3 indicated high risk, poor prognosis and a shorter OS time (*P* <0.05). Moreover, Kaplan-Meier survival curves showed that patients with high risk had significantly shorter OS time than those with low risk (*P <*0.05).

To verify the relationship between PTEN, PIK3C2A, ITPA and BCL3 expression with regard to OS and DFS, IHC analysis on tissue microarrays and Kaplan-Meier survival curves were performed. The results demonstrated that negative expression of PTEN and PIK3C2A were correlated with shorter OS and DFS time and worse prognosis, while positive expression of ITPA and BCL3 were related with shorter OS and DFS time and worse prognosis (*P <*0.05). These were consistent with the results from TCGA dataset, which also suggesting a prognostic prompt function of the four genes in ccRCC.

Furthermore, we analyzed the association of the four-gene signature with survival time according to both *SurvExpress* and IHC results. Our discovery suggested that the four-gene signature predicted survival better in ccRCC, indicating that the four-gene signature may be an independent predictor of prognosis in ccRCC. Univariate and multivariate Cox proportional hazard regression analysis were then conducted to verify the association of clinicopathological variables and the four genes’ expression levels with survival. Our results further testified that the risk (four-gene signature) was an independent prognostic factor of both OS and DFS (*P*<0.05).

However, some limitations were existed in our study. For example, only TCGA (KIRC) data set was selected for this research, resulting in limited samples for the four-gene signature model of prognosis. As a result, further verifying studies of our model in independent larger cohorts were required in the future.

In conclusion, our results suggested that the four-gene signature was related to the survival and was an independent predictor of prognosis in ccRCC. This may help to provide significant clinical implications for the prognosis prediction. However, the mechanisms of these genes impacting on the survival remain unknown. Therefore, further studies are needed to verify our findings and elucidate the molecular mechanisms so as to provide a deeper understanding of its function in predicting the prognosis of ccRCC.

## MATERIALS AND METHODS

### Datasets

**Figure 7:**
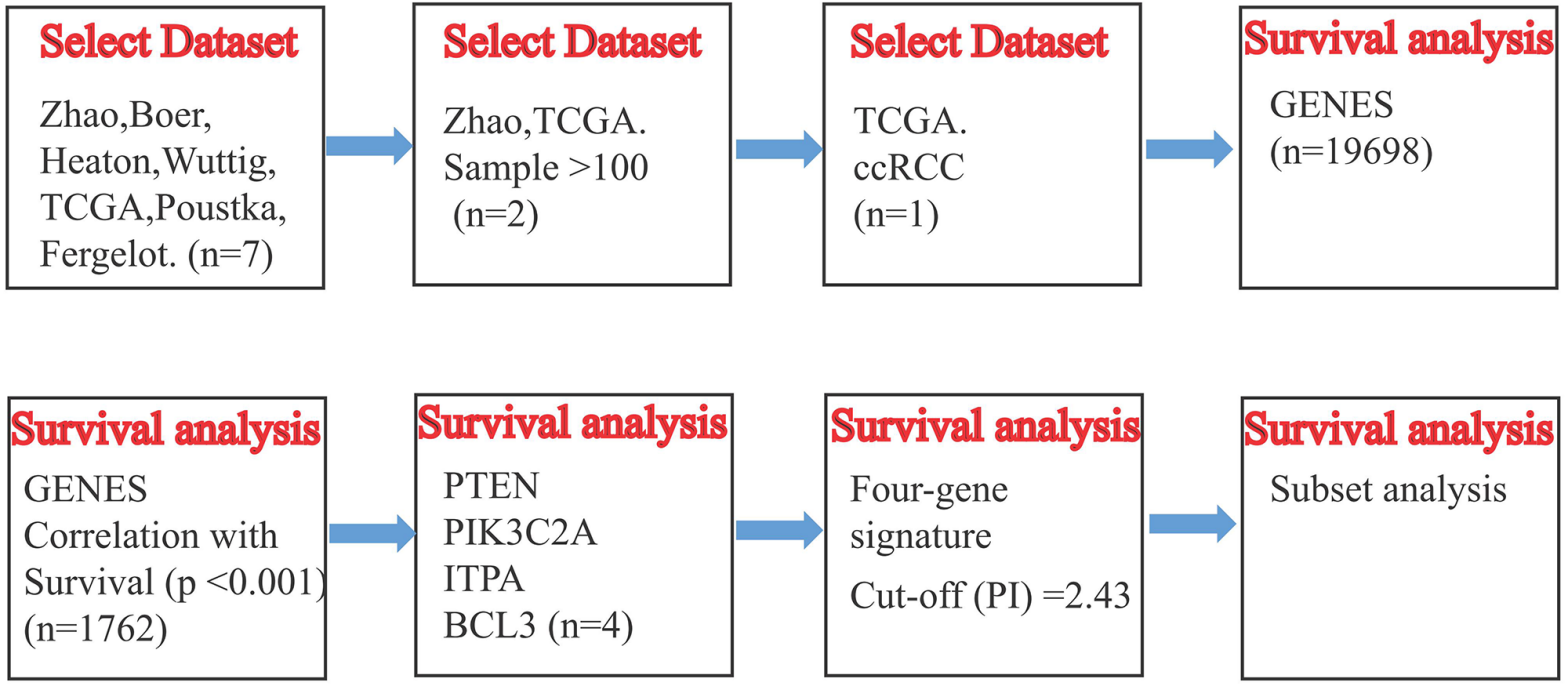
The procedure flow chart of SurvExpress Schematic overview of the procedure used in our study to construct the four-gene signature based on TCGA dataset.

In our analysis, *SurvExpress* was used to provide survival analysis and risk assessment. *SurvExpress* (http://bioinformatica.mty.itesm.mx/SurvExpress), which is a comprehensive gene expression database and online biomarker validation tool based on several datasets, can provide risk assessment and survival analysis in cancer datasets using a biomarker gene list as an input [40]. In databases provided by SurvExpress, TCGA (KIRC) and ZHAO database contain much larger samples (n> 100), and can provide more reliable results of survival analysis. However, RCC, but not ccRCC, is the study object of ZHAO, which does not match with our research. As a result, TCGA (KIRC) database was chose. Using this bioinformatic tool, we analyzed the expression differences of PTEN, PIK3C2A, ITPA, as well as BCL3, and their correlation with the survival of ccRCC patients in TCGA dataset (KIRC), and then analyzed the survival prognostic significance of the four-gene signature for ccRCC. PI, namely risk score, was often used for risk grouping [41, 42]. SurvExpress can perform risk grouping through two methods. The first method was default, which was to divide the ordered PI by the groups of risk so that the sample numbers of each group was equal. The second method was conducted through an optimization algorithm using the ordered PI. For example, log-rank test was accomplished using the arranged PI values for two risk groups. Then, the algorithm selected the dividing point, where *P* was at the minimum value. This process was extended to multi-groups tautologically to optimize a risk group till no change existed. The procedure flow chart of SurvExpress was as shown in Figure 7.

### Patients

In total, 174 cases of ccRCC tissues that were histopathologically diagnosed were collected from the Institute of Pathology of Tongji Hospital, Tongji Medical College of Huazhong University of Science and Technology from 2006 to 2009. Patients who had previously received chemotherapy and/or radiotherapy were excluded. The sex, age, tumor size and histological grade and clinical stage of the patients were collected. Then each clinical characteristic was grouped for subsequent analysis as follows: age (<55 years, ≥55 years); tumor size (<7cm, ≥7cm); Furhman grade (1, 2 *vs*. 3, 4) and clinical stage (I, II *vs*. III, IV) [43]. All tissues were collected under the highest ethical standards, and each patient provided written informed consent before randomization. Our research was a retrospective study with follow-up of patients on OS and DFS. OS was referred to the time from the first surgery to remove the tumor to death, regardless of any reason. DFS was referred to the time from the first surgery removing the tumor to disease recurrence / metastasis. SPSS software was used for all survival analysis. The adjusted hazard ratio (HR) as well as 95% confidence intervals (95% CI) was calculated by Cox proportional hazards model. In univariate analysis, variables that had p value less than 0.05 were used for multivariate analysis. Moreover, we also calculated the sensitivity and specificity of the gene signature.

### Tissue microarrays and IHC staining

Paraffin-embedded tissue microarrays were cut into 4-μm sections. After deparaffinization and hydration, the sections were pressure-cooked with antigen retrieval solution (10 mmol/L of sodium citrate buffer; pH 6.0) for 1.5 min. Then, 3% H_2_O_2_ was used to block endogenous peroxidase activity for 10 min. Subsequently, the sections were first incubated with protein blocker for 1 h at 37°C, and then incubated with anti-PTEN antibody (Ready-to-use, ZSGB-BIO, ZA-0251, China), anti-PIK3C2A antibody (1:50, Proteintech, 22028-1-AP, China), anti-ITPA antibody (1:50, Proteintech, 16134-1-AP, China) and anti-BCL3 antibody (1:50, Proteintech, 23959-1-AP, China) at 4°C overnight. After washing with PBS, the sections were incubated with anti-mouse IgG-HRP (1:500, Santa Cruz, CA, USA) for 1 h at 37°C, and stained using the Liquid DAB Substrate Kit (Invitrogen, Carlsbad, CA, USA). Finally, the sections were incubated with 3′, 3′-diaminobenzidine (Sigma, St Louis, MO, USA) and redyed with hematoxylin. All indicators were repeated twice.

### Evaluation of IHC

The IHC staining results were evaluated by two independent pathologists. Different criteria were used for proteins expressed in the cytoplasm and nuclei. For cytoplasmic proteins (PTEN, ITPA, PIK3C2A), the pathologists performed a semi-quantitative analysis according to the staining intensity and percentage of positive cells microscopically. We used the IHC integral criteria that had been used in many other studies [44, 45]. Staining intensity was scored as follow: “−” as 0 point; “+” as 1 point; “++” as 2 points; “+++” as 3 points. The percentage of positive cells (tumor cell counts ≥ 200 cells) was graded as follows: less than 25% as 0 point; 25%–50% as 1 point; 51%–75% as 2 points; 75%–100% as 3 points. The staining score was counted by adding the intensity score and the percentage score. A total score of 0–4 points was defined as negative expression, whereas 5–6 points were considered as positive expression. As nuclear proteins (BCL3), we referred to a separate IHC integral criterion regardless of intensity, because the protein had been reported to be located predominantly within the nucleus [46]. Each sample was composed of more than 200 tumor cells and the ratio of positive cells that were over 20% was considered to be BCL3 positive, while the rest were negative. Patients were divided into two groups based on the expression of PTEN, PIK3C2A, ITPA and BCL3. The low risk group coincided with at least three of PTEN (+), PIK3C2A (+), ITPA (-) and BCL3 (-), while the remaining was considered as high risk group. Detailed information was shown as Supplementary Table S1.

### Statistical analysis

The Chi-square test was used to compare between the two groups. To obtain and compare the survival curves, Kaplan-Meier method and log-rank test were performed [47]. The univariate and multivariate Cox proportional hazard regression analyses were conducted to evaluate independent prognostic factors associated with survival. All data were analyzed using SPSS 18.0 software, with the level of statistical significance at *P* < 0.05.

## SUPPLEMENTARY TABLE



## References

[1] Girgis H, Masui O, White NMA, Scorilas A, Rotondo F, Seivwright A, Gabril M, Filter ER, Girgis AHA, Bjarnason GA, Jewett MAS, Evans A, Al-Haddad S, Siu KWM, Yousef GM (2014). Lactate Dehydrogenase A is a potential prognostic marker in clear cell renal cell carcinoma. Mol Cancer.

[2] Lopez-Beltran A, Carrasco JC, Cheng L, Scarpelli M, Kirkali Z, Montironi R (2009). 2009 update on the classification of renal epithelial tumors in adults. Int J Urol.

[3] Siegel R, Naishadham D, Jemal A (2012). Cancer Statistics, 2012. Ca-Cancer J Clin.

[4] Park YH, Jung JW, Lee BK, Lee S, Jeong SJ, Byun SS, Lee SE (2015). Targeted therapy after complete resection of metastatic lesions in metastatic renal cell carcinoma. Int J Urol.

[5] Patard JJ, Leray E, Rodriguez A, Rioux-Leclercq N, Guille F, Lobel B (2003). Correlation between symptom graduation, tumor characteristics and survival in renal cell carcinoma. Eur Urol.

[6] Sun M, Lughezzani G, Perrotte P, Karakiewicz PI (2010). Treatment of metastatic renal cell carcinoma. Nat Rev Urol.

[7] Escudier B, Eisen T, Stadler WM, Szczylik C, Oudard S, Siebels M, Negrier S, Chevreau C, Solska E, Desai AA, Rolland F, Demkow T, Hutson TE, Gore M, Freeman S, Schwartz B (2007). Sorafenib in advanced clear-cell renal-cell carcinoma. The New England journal of medicine.

[8] Motzer RJ, Hutson TE, Tomczak P, Michaelson MD, Bukowski RM, Rixe O, Oudard S, Negrier S, Szczylik C, Kim ST, Chen I, Bycott PW, Baum CM, Figlin RA (2007). Sunitinib versus interferon alfa in metastatic renal-cell carcinoma. The New England journal of medicine.

[9] Creel PA (2014). Optimizing patient adherence to targeted therapies in renal cell carcinoma. Clinical journal of oncology nursing.

[10] Li J, Yen C, Liaw D, Podsypanina K, Bose S, Wang SI, Puc J, Miliaresis C, Rodgers L, McCombie R, Bigner SH, Giovanella BC, Ittmann M, Tycko B, Hibshoosh H, Wigler MH (1997). PTEN, a putative protein tyrosine phosphatase gene mutated in human brain, breast, and prostate cancer. Science.

[11] Kluth M, Runte F, Barow P, Omari J, Abdelaziz ZM, Paustian L, Steurer S, Tsourlakis MC, Fisch M, Graefen M, Tennstedt P, Huland H, Michl U, Minner S, Sauter G, Simon R (2015). Concurrent deletion of 16q23 and PTEN is an independent prognostic feature in prostate cancer. Int J Cancer.

[12] Mithal P, Allott E, Gerber L, Reid J, Welbourn W, Tikishvili E, Park J, Younus A, Sangale Z, Lanchbury JS, Stone S, Freedland SJ (2014). PTEN loss in biopsy tissue predicts poor clinical outcomes in prostate cancer. Int J Urol.

[13] Lee HJ, Lee HY, Lee JH, Lee H, Kang G, Song JS, Kang J, Kim JH (2014). Prognostic Significance of Biallelic Loss of PTEN in Clear Cell Renal Cell Carcinoma. J Urology.

[14] Zhu CY, Wei JX, Tian X, Li Y, Li XD (2015). Prognostic role of PPAR-gamma and PTEN in the renal cell carcinoma. Int J Clin Exp Patho.

[15] Manning BD, Cantley LC (2007). AKT/PKB signaling: Navigating downstream. Cell.

[16] Vivanco I, Sawyers CL (2002). The phosphatidylinositol 3-kinase-AKT pathway in human cancer. Nat Rev Cancer.

[17] Biswas K, Yoshioka K, Asanuma K, Okamoto Y, Takuwa N, Sasaki T, Takuwa Y (2013). Essential role of class II phosphatidylinositol-3-kinase-C2alpha in sphingosine 1-phosphate receptor-1-mediated signaling and migration in endothelial cells. J Biol Chem.

[18] Karakas B, Bachman KE, Park BH (2006). Mutation of the PIK3CA oncogene in human cancers. British journal of cancer.

[19] Chakraborty S, Mohiyuddin SM, Gopinath KS, Kumar A (2008). Involvement of TSC genes and differential expression of other members of the mTOR signaling pathway in oral squamous cell carcinoma. BMC cancer.

[20] Poppe B, Vandesompele J, Schoch C, Lindvall C, Mrozek K, Bloomfield CD, Beverloo HB, Michaux L, Dastugue N, Herens C, Yigit N, De Paepe A, Hagemeijer A, Speleman F (2004). Expression analyses identify MLL as a prominent target of 11q23 amplification and support an etiologic role for MLL gain of function in myeloid malignancies. Blood.

[21] Smid A, Karas-Kuzelicki N, Milek M, Jazbec J, Mlinaric-Rascan I (2014). Association of ITPA Genotype with Event-Free Survival and Relapse Rates in Children with Acute Lymphoblastic Leukemia Undergoing Maintenance Therapy. Plos One.

[22] Menezes MR, Waisertreiger ISR, Lopez-Bertoni H, Luo X, Pavlov YI (2012). Pivotal Role of Inosine Triphosphate Pyrophosphatase in Maintaining Genome Stability and the Prevention of Apoptosis in Human Cells. Plos One.

[23] Shichijo S, Azuma K, Komatsu N, Kawamoto N, Takedatsu H, Shomura H, Sawamizu H, Maeda Y, Ito M, Itoh K (2003). Identification of two novel tumor-associated antigens recognized by HLA-B46-restricted cytotoxic T lymphocytes. Int J Mol Med.

[24] John T, Black MA, Toro TT, Leader D, Gedye CA, Davis ID, Guilford PJ, Cebon JS (2008). Predicting clinical outcome through molecular profiling in stage III melanoma. Clin Cancer Res.

[25] Maldonado V, Melendez-Zajgla J (2011). Role of Bcl-3 in solid tumors. Mol Cancer.

[26] Brenne AT, Fagerli UM, Shaughnessy JD, Vatsveen TK, Ro TB, Hella H, Zhan FH, Barlogie B, Sundan A, Borset M, Waage A (2009). High expression of BCL3 in human myeloma cells is associated with increased proliferation and inferior prognosis. Eur J Haematol.

[27] Wakefield A, Soukupova J, Montagne A, Ranger J, French R, Muller WJ, Clarkson RWE (2013). Bcl3 Selectively Promotes Metastasis of ERBB2-Driven Mammary Tumors. Cancer Res.

[28] Puvvada SD, Funkhouser WK, Greene K, Deal A, Chu HT, Baldwin AS, Tepper JE, O'Neil BH (2010). NF-kappa B and Bcl-3 Activation Are Prognostic in Metastatic Colorectal Cancer. Oncology.

[29] Dimitrakopoulos FID, Antonacopoulou AG, Kottorou A, Marousi S, Koukourikou I, Kalofonou M, Panagopoulos N, Scopa C, Dougenis D, Papadaki H, Papavassiliou AG, Kalofonos HP (2015). Variant of BCL3 gene is strongly associated with five-year survival of non-small-cell lung cancer patients. Lung Cancer.

[30] Kashatus D, Cogswell P, Baldwin AS (2006). Expression of the Bcl-3 proto-oncogene suppresses p53 activation. Gene Dev.

[31] Ahmed SU, Milner J (2009). Basal Cancer Cell Survival Involves JNK2 Suppression of a Novel JNK1/c-Jun/Bcl-3 Apoptotic Network. Plos One.

[32] Lim HJ, Crowe P, Yang JL (2015). Current clinical regulation of PI3K/PTEN/Akt/mTOR signalling in treatment of human cancer. Journal of cancer research and clinical oncology.

[33] Urban BC, Collard TJ, Eagle CJ, Southern SL, Greenhough A, Hamdollah-Zadeh M, Ghosh A, Poulsom R, Paraskeva C, Silver A, Williams AC (2016). BCL-3 expression promotes colorectal tumorigenesis through activation of AKT signalling. Gut.

[34] Ljungberg B, Cowan NC, Hanbury DC, Hora M, Kuczyk MA, Merseburger AS, Patard JJ, Mulders PFA, Sinescu IC (2010). EAU Guidelines on Renal Cell Carcinoma: The 2010 Update. Eur Urol.

[35] Park I, Lee JL, Ahn JH, Lee DH, Lee KH, Jeong IG, Song C, Hong B, Hong JH, Ahn H (2014). Active surveillance for metastatic or recurrent renal cell carcinoma. Journal of cancer research and clinical oncology.

[36] Gulati S, Martinez P, Joshi T, Birkbak NJ, Santos CR, Rowan AJ, Pickering L, Gore M, Larkin J, Szallasi Z, Bates PA, Swanton C, Gerlinger M (2014). Systematic Evaluation of the Prognostic Impact and Intratumour Heterogeneity of Clear Cell Renal Cell Carcinoma Biomarkers. Eur Urol.

[37] Wu XW, Weng LH, Li XJ, Guo C, Pal SK, Jin JM, Li YP, Nelson RA, Mu B, Onami SH, Wu JJ, Ruel NH, Wilczynski SP, Gao HL, Covarrubias M, Figlin RA (2012). Identification of a 4-microRNA Signature for Clear Cell Renal Cell Carcinoma Metastasis and Prognosis. Plos One.

[38] Ge YZ, Wu R, Xin H, Zhu M, Lu TZ, Liu H, Xu Z, Yu P, Zhao YC, Li MH, Hu ZK, Zhao Y, Zhong B, Xu X, Zhou LH, Xu LW (2015). A tumor-specific microRNA signature predicts survival in clear cell renal cell carcinoma. Journal of cancer research and clinical oncology.

[39] Wang Z, Xie H, Zhou L, Liu Z, Fu H, Zhu Y, Xu L, Xu J (2016). CCL2/CCR2 axis is associated with postoperative survival and recurrence of patients with non-metastatic clear-cell renal cell carcinoma. Oncotarget.

[40] Aguirre-Gamboa R, Gomez-Rueda H, Martinez-Ledesma E, Martinez-Torteya A, Chacolla-Huaringa R, Rodriguez-Barrientos A, Tamez-Pena JG, Trevino V (2013). SurvExpress: An Online Biomarker Validation Tool and Database for Cancer Gene Expression Data Using Survival Analysis. Plos One.

[41] Akter S, Choi TG, Nguyen MN, Matondo A, Kim JH, Jo YH, Jo A, Shahid M, Jun DY, Yoo JY, Nguyen NN, Seo SW, Ali L, Lee JS, Yoon KS, Choe W (2015). Prognostic value of a 92-probe signature in breast cancer. Oncotarget.

[42] Nguyen MN, Choi TG, Nguyen DT, Kim JH, Jo YH, Shahid M, Akter S, Aryal SN, Yoo JY, Ahn YJ, Cho KM, Lee JS, Choe W, Kang I, Ha J, Kim SS (2015). CRC-113 gene expression signature for predicting prognosis in patients with colorectal cancer. Oncotarget.

[43] Park JH, Lee C, Suh JH, Chae JY, Kim HW, Moon KC (2015). Decreased ARID1A expression correlates with poor prognosis of clear cell renal cell carcinoma. Hum Pathol.

[44] Dong M, Wang HY, Zhao XX, Chen JN, Zhang YW, Huang Y, Xue L, Li HG, Du H, Wu XY, Shao CK (2016). Expression and prognostic roles of PIK3CA, JAK2, PD-L1 and PD-L2 in EBV-associated gastric carcinoma. Hum Pathol.

[45] Loupakis F, Pollina L, Stasi I, Ruzzo A, Scartozzi M, Santini D, Masi G, Graziano F, Cremolini C, Rulli E, Canestrari E, Funel N, Schiavon G, Petrini I, Magnani M, Tonini G (2009). PTEN Expression and KRAS Mutations on Primary Tumors and Metastases in the Prediction of Benefit From Cetuximab Plus Irinotecan for Patients With Metastatic Colorectal Cancer. Journal Of Clinical Oncology.

[46] Schlette E, Rassidakis GZ, Canoz O, Medeiros LJ (2005). Expression of bcl-3 in chronic lymphocytic leukemia correlates with trisomy 12 and abnormalities of chromosome 19. American journal of clinical pathology.

[47] Shahid M, Cho KM, Nguyen MN, Choi TG, Jo YH, Aryal SN, Yoo JY, Yun HR, Lee JW, Eun YG, Lee JS, Kang I, Ha J (2016). Prognostic value and their clinical implication of 89-gene signature in glioma. Oncotarget.

