# Supplementary material for: A four-gene signature predicts survival in clear-cell renal-cell carcinoma
Source: Oncotarget. 2016 Oct 13;7(50):82712–26. doi: 10.18632/oncotarget.12631 (PMC5347726; doi:10.18632/oncotarget.12631)
Supplement: Supplementary file 1 [file oncotarget-07-82712-s001.pdf]

A four-gene signature predicts survival in clear-cell renal-cell carcinoma

SUPPLEMENTARY TABLE

Supplementary Table S1:

PTEN	PIK3C2A	ITPA	BCL3	Risk
+	+	-	-	Low
+	-	-	-	Low
+	+	+	-	Low
+	+	-	+	Low
-	+	-	-	Low
+	+	+	+	High
+	-	+	-	High
+	-	-	+	High
-	+	+	-	High
-	+	-	+	High
-	-	-	-	High
+	-	+	+	High
-	+	+	+	High
-	-	-	+	High
-	-	+	-	High
-	-	+	+	High
